# The Delivery and Activation of Growth Factors Using Nanomaterials for Bone Repair

**DOI:** 10.3390/pharmaceutics15031017

**Published:** 2023-03-22

**Authors:** Yiwei Li, Chun Xu, Chang Lei

**Affiliations:** 1 Australian Institute for Bioengineering and Nanotechnology, The University of Queensland, St Lucia, QLD 4072, Australia; 2 School of Dentistry, The University of Queensland, Brisbane, QLD 4006, Australia

**Keywords:** nanomaterials, growth factors, bone regeneration, bone defect, bone graft

## Abstract

Bone regeneration is a comprehensive process that involves different stages, and various growth factors (GFs) play crucial roles in the entire process. GFs are currently widely used in clinical settings to promote bone repair; however, the direct application of GFs is often limited by their fast degradation and short local residual time. Additionally, GFs are expensive, and their use may carry risks of ectopic osteogenesis and potential tumor formation. Nanomaterials have recently shown great promise in delivering GFs for bone regeneration, as they can protect fragile GFs and control their release. Moreover, functional nanomaterials can directly activate endogenous GFs, modulating the regeneration process. This review provides a summary of the latest advances in using nanomaterials to deliver exogenous GFs and activate endogenous GFs to promote bone regeneration. We also discuss the potential for synergistic applications of nanomaterials and GFs in bone regeneration, along with the challenges and future directions that need to be addressed.

## 1. Introduction

The repairing of large bone defects remains a significant challenge in the clinic [1]. Over 2 million bone grafting surgeries are performed worldwide each year to restore the functions of defects [2], and the global market for bone grafting materials is expected to reach USD 1.4 billion by 2025 [3]. Among various grafting materials, autologous bone grafts have ideal physiologic properties and have been used for centuries [4]. Growth factors (GFs) that favor bone growth were also detected in fresh autologous bone grafts [5]; however, due to the limited resource of autologous bone and the risk of donor site morbidity [6], the development of synthetic bone graft substitutes for repairing bone is highly demanded. The ideal synthetic material should have good biocompatibility, excellent osteogenic as well as angiogenic activities, suitable mechanical properties, and an affordable cost [7]. With advances in material synthesis, many biomaterials have been developed as bone graft substitutes, including metals (iron, magnesium, alloy, etc.), ceramics (bioglass, calcium carbonate, gypsum, etc.) and polymers (collagen, chitosan, fibrin, etc.) [4,8]. Among them, biomaterials with nanostructures have attracted great interest in recent years as they enable the reassembly of new bone at the nanoscale, which is closer to the natural bone structure [9]. Specifically, the small nano-sizes, large surface areas, tunable surface chemistries, and porous structures of nanomaterials make them suitable for molecule (e.g., GFs and drugs) loading and delivery. Many nanomaterials have been used for bone regeneration, such as hydroxyapatite (HA) nanoparticles [10,11,12,13,14], silicon-based nanomaterials [15], carbon-based nanomaterials [16], graphene nanomaterials [17,18], and metal-based (gold, silver, platinum, iron, etc.) nanomaterials [19]. It is important to note that the nano–bio interactions that occur at the cellular, molecular, and atomic levels have significant implications for the safety and efficacy of nanomaterials in various biomedical applications, including drug delivery and tissue engineering.

Bone regeneration is a complex process that requires the coordination of different cells and signal pathways. It involves phases of inflammation, angiogenesis, and tissue healing [20]. As essential biomolecules present in all stages of bone regeneration, GFs stimulate osteogenesis by activating key genes and transcription factors or promoting osteoblasts’ differentiation [21]. In general, the whole process of bone regeneration is directly influenced by bone morphogenetic protein (BMP) [22] and transforming growth factor-beta (TGF-β) [23]. BMP-2, BMP-4, and BMP-7, from the BMP family, have been found to be osteogenic inducers [24], and the newly discovered BMP-9 has also attracted strong interest from scientists [25]. In the process of an inflammatory response, macrophages polarize to an M2 phenotype and release GFs, including interleukins (ILs) [26], fibroblast growth factors (FGFs) [27], and tumor necrosis factors (TNFs) [28], all of which have the function of inducing osteoblast migration [29]. During angiogenesis, vascular endothelial growth factor (VEGF) [30] and platelet-derived growth factor (PDGF) [31] play essential roles in the induction of the formation of new blood vessels. The main GFs involved in the tissue healing phase are FGFs and PDGF for fibroblast stimulation [32].

There are many studies that utilize GFs for bone defect treatment via either exogenous GF delivery or endogenous GF stimulation. It is difficult for exogenous GFs to function without a carrier due to the following reasons: The first is their short half-life [33], which requires repeated administration or an increased dosage to be overcome. The excessive use of BMP-2 has been shown to cause uncontrollable bone regeneration and cancer [34,35]. Although recombinant human bone morphogenetic proteins (rhBMPs) have been approved by the FDA for clinical use [36], the amount used in surgery is often a hundred times greater than that seen under normal conditions and could lead to uncontrollable consequences. Another critical reason is the biological instability under thermal or fluctuant pH conditions [37]. Without protection, in the specific microenvironment of defects natural GFs usually degrade rapidly and are unable to reach the cellular matrix, where they can function. Thirdly, controlled and targeted delivery is unachievable, which significantly hinders the function of GFs [38]. Therefore, using carriers to protect and achieve the controlled delivery of GFs is necessary [39]. As mentioned above, nanomaterials are popular carriers of GFs because of their unique nanoscale properties. The large surface areas and porous structures of nanomaterials facilitate the high loading of GFs, and their easy surface modification enables the targeted delivery of GFs. In turn, the addition of GFs can improve the osteogenic activity of nanomaterials. Therefore, nanomaterial-based exogenous GF delivery is a promising strategy for achieving satisfactory bone repairing outcomes.

Activating endogenous GFs to improve the body’s self-healing ability is another strategy. It usually induces lower immune rejection or resistance in patients compared to that of exogenous GF delivery. Nanomaterials are found to activate endogenous GFs via immune system stimulation, the stimulation of signal pathways, or gene therapy. The morphologies, surface chemistries, and contents of nanomaterials are found to modulate immune cells or signal pathways to release GFs for bone repairing [40,41,42]. Nanomaterials can also deliver genes and small-molecule drugs to stimulate the production of endogenous GFs and protect them from digestion [43].

**Figure 1:**
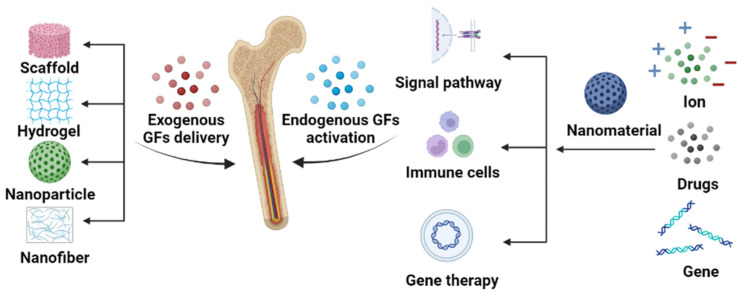
Schematic illustration of the synergistic effect of nanomaterials and GFs on bone repair. The roles of nanomaterials include exogenous GF delivery and endogenous GF stimulation.

**Figure 2:**
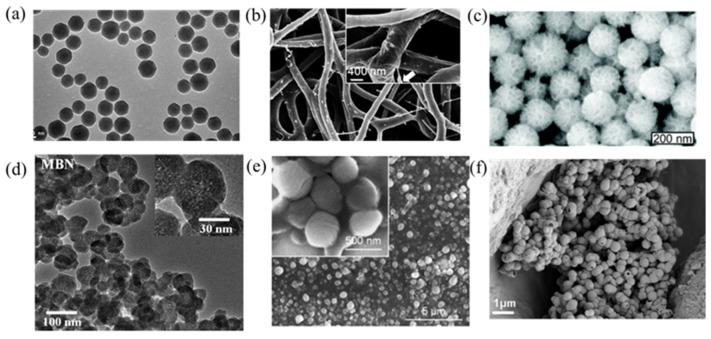
Images of various nanoparticles introduced in this review. (**a**) Mesoporous silica nanoparticles (MSNs) used for bFGF delivery [48]. (**b**) SF/PCL/PVA nanofibers used for dual GF delivery [49]. (**c**) Polyethylenimine-modified porous silica nanoparticles (PPSNs) used for pBMP-2 delivery [50]. (**d**) Magnetic iron oxide nanomaterials (MBNs) used for the stimulation of GF-related signal pathways [51]. (**e**) Deferoxamine@poly (ε-caprolactone) nanoparticles (DFO@PCL NP) used for the stimulation of macrophage polarization [52]. (**f**). RNA-activated matrices lipopolyplex (RAM-LPR) for miRNA delivery [53].

Utilizing GFs in the treatment of bone repairing is an attractive area, and several reviews have been published in recent years that discuss either GF- or nanomaterial-based bone and tissue regeneration [44,45,46,47]. In this review we discuss the integration of nanomaterials with GFs for bone regeneration, including exogenous GF delivery and endogenous GF activation (Figure 1). This review aims to elucidate the relationship between the properties of nanomaterials (Figure 2) and GFs in terms of bone regeneration, as well as providing an outlook on designing advanced nanomaterials with better GF utilization for bone defect treatment.

## 2. Exogenous Growth Factor Delivery

In the early days of GF therapy in bone defect treatment, GFs were often used via local direct injections, which were a very underutilized method due to the short half-life and unstable nature of GFs in human circulation [54]. To achieve the desired therapeutic effect, large doses of GFs were required, which led to high costs and severe side effects. Therefore, sustained release and local delivery strategies with which to increase the efficiency of GF utilization have become significant research areas in bone regeneration. As mentioned above, many nanomaterials have been developed as delivery platforms of GFs that are cost-effective and enable controlled release. GFs are usually immobilized to the surface of nanomaterials or loaded into their interior void/pores via physical adsorption or chemical bonding [45]. To date, the nanomaterials used for delivering GFs in bone regeneration have been molded into four forms: scaffolds, hydrogels, nanofibers, and nanoparticles (Figure 1).

### 2.1. Scaffolds

A scaffold is a prevalent form of a bone graft, with a three-dimensional structure and high porosity, allowing cells to proliferate, migrate, and differentiate [55]. Nanocomposite scaffolds can be easily obtained by mixing nanomaterials into the raw material before scaffold fabrication [56] or by loading nanomaterials into pre-formed scaffolds [57]. The incorporation of nanomaterials into scaffolds can improve their mechanical properties and create a nanostructure with an enhanced surface area as well as roughness, promoting cell adhesion and proliferation on the scaffold [58]. In addition, it is often difficult to achieve satisfactory loading efficiencies of solely GFs in scaffolds. By incorporating GFs into nanomaterials and then integrating them into scaffolds, their loading efficiencies could be significantly improved, leading to increased osteogenic activity [45]. Furthermore, a careful design of the nano–bio interface can create a favorable environment for GFs, allowing for the controlled release and sustained delivery of the factors over an extended period [59,60,61]. This approach also provides protection to GFs, leading to increased osteogenic activity.

**Figure 3:**
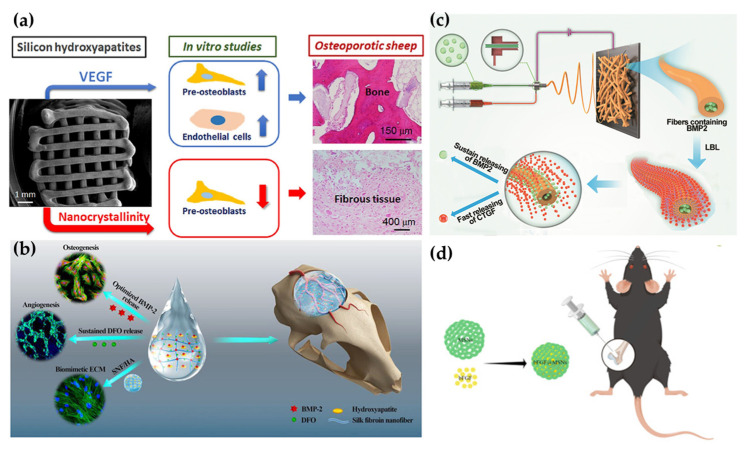
Exogenous growth factors delivered by various types of nanomaterials. (**a**). VEGF-adsorbed nano-SiHA scaffolds enhanced bone regeneration in an osteoporotic sheep model [12]. (**b**). An injectable hydrogel consisting of SNF and HA for DFO and BMP-2 delivery [64]. (**c**). A core–shell SF/PCL/PVA nanofibrous mat for the controlled release of dual GFs: BMP-2 and CTGF [49]. (**d**). MSN-based bFGF delivery for enhancing bone regeneration [48].

The delivery of GFs through scaffolds has been shown to be an effective strategy for enhancing osteogenic capacity, as demonstrated in numerous studies. As illustrated in Figure 3a, a scaffold fabricated with silicon-substituted hydroxyapatite (SiHA) nanocrystals, doped with VEGF, has been demonstrated to induce the differentiation of MC3T3 cells (preosteoblastic) and promote new bone formation in a sheep model [12]. In another study, the same research group developed SiHA/VEGF-coated titanium alloy (Ti6Al4V-ELI) scaffolds for bone repair, which demonstrated good compatibility between nanomaterials and conventional metal bone implants [62]. In addition to the role of carriers, nanomaterials also provide protection to GFs. Y. Zhang et al. prepared a novel nano-dressing by loading BMP-2 into a metal–phenolic network (MPN), which allows the slow release of BMP-2 through a physical barrier effect. This nano-dressing was coated to the surface of a porous poly(dl-lactide) (PPLA) scaffold to achieve stem cell recruitment and differentiation [63].

Compared to single GF delivery, dual GF delivery has been shown to result in improved outcomes for bone repair due to the synergistic effect of two GFs. One simple method for incorporating GFs into scaffolds is through physical adsorption. Kuttappan et al. prepared two dual GF delivery systems by loading VEGF+BMP-2 or FGF2+BMP-2 into a nanocomposite fibrous scaffold (CS) through physical adsorption. Both of the systems continuously released GFs for 30 days and demonstrated similar excellent osteogenic as well as angiogenic capacities in Wistar rats [65]. Wang et al. fabricated a scaffold by encapsulating chitosan oligosaccharide/heparin nanoparticles (CSO/H NPs) into chitosan-agarose-gelatin. This scaffold was further loaded with SDF-1 and BMP-2 in a high loading efficiency (>80%) of both of the two GFs and achieved a slow release over 15 days, both in vitro and in vivo [66]. Another approach is to mix GFs or GF-loaded nanomaterials into a solution and then form a scaffold using methods such as lyophilization, thermally induced phase separation, or particulate leaching [67]. These methods enable the integration of GFs or GF-loaded nanomaterials into the scaffold structure. In a study published in *Biomaterials*, BMP-2 and TGF-β1 were mixed with a silk protein (SF) fibroin/carbon nanofiber (CNF)-containing solution and lyophilized into a scaffold. The average entrapment efficiency of these two GFs was 58.67%, which had a slow release period of up to 42 days [68]. Similarly, by means of lyophilization, rhBMP-2 and rhTGF-β3 were loaded into an SF/nHA scaffold that mimics the structure of cartilage. The scaffold exhibited a 30-day sustained release of GFs while significantly reducing sudden release within 24 h [69]. Using 3D printing technology, a scaffold was prepared by loading CTGF and TGF-β3 onto polydopamine nanoparticles through covalent binding. The resulting scaffold had a mechanical strength comparable to that of a natural disc and was able to sustainably release approximately 60% of the loaded GFs over a period of 35 days. Significant new bone formation had been found in nude mice [70].

Recently, there have been many studies that report on the co-delivery of GF and chemical agents (e.g., GF stimulants). Yao et al. developed a novel scaffold by incorporating BMP-2-loaded mesoporous silicate nanoparticles (MSNs) into a nanofibrous gelatin, after which they cross-linked by adding desferrioxamine (DFO). DFO is a GF stimulator that mimics the hypoxic environment to activate hypoxia-inducible factor 1 alpha (HIF-1α). Under the protection of nanoparticles, both BMP2 and DFO were released continuously over 28 days. An in vitro study showed that the scaffold significantly improved the expression of VEGF and ALP activity [71]. In another work, a scaffold that contained VEGF and L-ascorbic acid 2-phosphate also demonstrated excellent angiogenic capacity [72].

### 2.2. Hydrogels

Hydrogels, such as collagen, hyaluronic acid, and elastin, have excellent biocompatibility [73] and mimic the extracellular matrix (ECM) [74] to provide cues for cell differentiation [75], but often lack osteogenic activity. By incorporating nanomaterials into hydrogels [76], the composite hydrogels can achieve higher GF loading as well as exhibit controlled drug release behavior, enhanced surface interactions with biological entities [77], and improved mechanical properties [78], in addition to specific physical properties from the nanomaterials, such as magnetic and thermal conductivity [79]. There are two common ways of combining hydrogels and nanomaterials: directly mixing nanomaterials with hydrogels or incorporating hydrogels into a preformed nanomaterial-based scaffold.

Various organic nanoparticles have been used to prepare nanocomposite hydrogels, which more substantially ensure the biocompatibility of the implant material. An organic nanoparticle made from chitosan and carboxymethyl chitosan was used to load SDF-1α through electrostatic adsorption and was encapsulated in a chitosan/β-glycerol phosphate disodium salt hydrogel. Compared to the direct loading of SDF-1α, which resulted in an 85% release within the first four days, the SDF-1α protected by nanoparticles showed an excellent slow release, with approximately 40% SDF-1α released over 28 days [80]. The combination of GFs and nanomaterials can also occur through chemical bonding in addition to physical electrostatic adsorption. Yuan et al. incorporated beta-defensin 2 (BD2) into a bionic nanofibrous hybrid hydrogel composed of bacterial cellulose and calcium ions through interacting with alginates [81]. The sustained release of BD2 caused the hydrogel to exhibit excellent antibacterial activity, as well as angiogenic and osteogenic capabilities. The mechanical properties of the hydrogel were also improved, providing physical support for bone repair.

Inorganic nanomaterials are also widely used in nanocomposite hydrogels due to their ease of synthesis and unique physical characteristics. Two-dimensional black phosphorus nanosheets (BPNSs) with VEGF modifications were incorporated into dynamic DNA hydrogels, forming a nanophysical network with excellent mechanical strength. Results from a rat skull defect model showed that the hydrogel synergistically promoted osteogenesis and vascular growth [82]. Magnetic inorganic nanoparticles, glycosylated superparamagnetic iron oxide nanoparticles, were used to encapsulate BMP-2 and incorporate it into the hydrogel. An external magnetic field could be applied to align and arrange the nanoparticles within the hydrogel in a gradient formation, providing a spatial orientation for osteogenesis through the release of BMP-2 with varying concentration gradients [83]. A hollow gold nanoparticle (HGNP)-incorporated hydrogel was developed to achieve the controlled release of BMP-2 at specific time points by triggering HGNP via using an NIR laser [84]. In addition to their physical properties, the exceptional surface chemistry of the nanomaterials also enhances the hydrogel’s osteogenic activity. Nanographene oxide (nGO) has been added to hydrogels to utilize its high protein loading efficiency. In a study, nGO was able to bind up to 90.5% of TGF-β3 at a concentration of 0.5 µg/mL. The incorporation of nGO into hydrogels significantly decreased the rapid release of GF and improved in vitro cartilage formation in human-bone-marrow-derived stem cells (hBMSCs) [85]. Nanodiamonds, another carbon-based material, was used for VEGF loading and showed sustained VEGF release without causing an inflammatory response [86].

Organic–inorganic hybrid nanomaterials have become a new strategy for preparing nanocomposite hydrogels. Figure 3b shows an implantable hydrogel composed of DFO-loaded organic silk fibroin nanofiber (SNF) and BMP-2-loaded inorganic HA nanoparticles, with a 4:6 *wt*/*wt* ratio of SNF to HA, similar to the organic–inorganic ratio in natural bone. The hydrogel exhibited strong osteogenic as well as angiogenic capabilities and demonstrated a slow release of both DFO and BMP-2 for over 40 days via in vitro experiments [64]. In another study, an organic–inorganic nanoparticle, polyhedral oligomeric silsesquioxane (POSS), was incorporated into hydrogels to create a porous structure and increase the stiffness. The POSS-loaded hydrogel was employed to deliver BMP-2 and VEGF to promote new bone production [87].

The hydrogel has excellent biocompatibility, making it suitable for incorporating cells to create a bioink for 3D bioprinting. A bioink contains VEGF-loaded nanoclay as well as hBMSC cells, and was found to promote a high degree of angiogenesis in a chick chorioallantoic membrane model [88]. The incorporation of cells into the hydrogel composites makes them increasingly biomimetic, mimicking the spatial and temporal release of growth factors during bone repair with the help of nanomaterials. In the future, scientists aim to develop hydrogels with even more complex physiological cues that guide cell differentiation and better imitate the ECM.

### 2.3. Nanofibers

Nanofibers are also a form of implant widely used for bone regeneration that have high porosity and pore connectivity to mimic ECM structure [89]. The ECM acts as a GF reservoir and facilitates bone regeneration by releasing GFs during the bone repair process. Scientists aim to mimic this process by loading GFs into nanofibers and manipulating the related nano–bio interaction to enhance bone regeneration.

Electrostatic spinning is a commonly used method to prepare nanofibers. As an example, an artificial periosteum was created by combining electrostatically spun polylactic acid (PLLA) nanofibers with hyaluronic acid and encapsulating them with VEGF. The periosteum was able to slowly release VEGF over 4 weeks, leading to the acceleration of early angiogenesis and increased stimulation of osteoblast adhesion, promoting the process of bone regeneration [90]. In another study, an electrostatic spinning method was used to produce a PCL/PLGA nanofiber doped with TGF-β3, which was shown to promote membrane-derived stem cell (SDSC)-mediated fibrocartilage regeneration [91]. In addition to incorporating individual GFs, a study has reported the loading of dual GFs (FGF-2 and BMP-2) onto an affinity surface-modified electrospun gelatin nanofiber. This significantly increased the expression of osteogenic genes, such as RunX2, COL1α1, and OCN, demonstrating the potential of using dual GF-loaded nanofibers to enhance bone regeneration [92]. The electrostatic spinning technique can also be combined with layer-by-layer (LBL) technology to produce nanofibers, allowing for the creation of multifunctional coatings that incorporate proteins, genes, and other biomolecules. As shown in Figure 3c, SF/PCL/PVA nanofibers loaded with different GFs were fabricated to load different GFs and achieve varying release rates. CTGF was loaded onto the surface for rapid release by using LBL technology, while BMP-2 was encapsulated in the core for sustained release, mimicking the natural physiological process of bone repair. This system resulted in increased new bone formation and angiogenesis in a rat cranial defect model compared to a single BMP-2-loaded control [49]. In other studies, dopamine (PDA) was used as a coating for dual GF loading. The presence of PDA led to a significant increase in the loading of VEGF and BMP-2 on electrostatically spun PLLA nanofibrous membranes, which were then shown to improve femoral and periosteal defects in rats [93].

### 2.4. Nanoparticles

The nanoparticle-based delivery of growth factors (GFs) has emerged as a promising approach for promoting bone regeneration. The use of nanomaterials and manipulating their interactions with biomolecules/biosystems offer numerous benefits, including enhanced biocompatibility, the improved stability of GFs, and the controlled release of GFs for optimal therapeutic effects, making them an attractive strategy for treating bone injuries and promoting bone repair.

Inorganic nanomaterials, favored for their simple synthesis, are popular in the field of bone repair, with nano-hydroxyapatite (nHA) being the most widely used. nHA, chemically similar to human bone, promotes bone growth through its effect on osteoblasts. The combination of nHA and GF has been proven to be an effective method for bone regeneration in many studies. For instance, a study showed that the combination of rh-BMP2 with nHA microspheres composed of nanowhiskers successfully restored a femoral defect in rats within eight weeks [94]. In another study, nHA was added to a slow-release polymer, polylactic acid-polyethylene glycol (PLA-PEG), and achieved the sustained release of BMP-2 for three weeks, resulting in a substantial fusion of a spinal defect in rats. The nHA was completely resorbed and replaced by new bone tissue, showcasing the potential of this approach for bone repair [10]. GFs such as rhVEGF [95] have also been used in combination with nHA to promote bone regeneration.

In addition to nHA, silica-based nanoparticles have been demonstrated to have potential as drug delivery platforms for promoting osteogenesis due to the osteogenic as well as angiogenic properties of silica ions [96]. One of the most commonly used types of silica-based nanoparticles is MSNs, which are highly biocompatible and have a large surface area, making them ideal for delivering bioactive molecules. A study that loaded bFGF into MSNs to achieve sustained release showed that this composite promoted osteogenesis and stimulated the Wnt/β-catenin signal pathway [48]. The rich surface areas and porous structures of MSNs allow them to carry more GFs or other small molecules simultaneously. Dentin matrix extract protein (DMEP) was identified as a complex GF mixture, and MSNs loaded with DMEP were demonstrated to promote cartilage regeneration in vitro and in vivo [97]. To mitigate the risk of infection in defective bone tissue, a study showed that co-loading the antibiotic cefazolin with BMP-2 into MSNs significantly increased new bone formation and reduced the release of inflammatory factors IL-1 and IL-4 in a mouse fracture model [98]. The flexible surface of MSNs also provides alternative loading methods for growth factors. Zhou et al. grafted BMP-2 peptides onto MSNs’ surfaces by modifying amine groups on their surfaces, and also loaded a bone marrow stromal cell inducer, DEX, inside the MSNs. The transfection efficiency of the nanoparticles was significantly increased, and the combination of BMP-2 and DEX synergistically enhanced osteogenic activity in addition to promoting ectopic osteogenesis in mice [99].

Carbon-based nanoparticles have been studied for their unique properties, such as their biocompatibility, high surface area, and high mechanical strength, which make them promising candidates for use in bone repair applications [100]. Nano-graphene oxide (nGO) was coated onto the surface of Fe_3_O_4_ magnetic nanoparticles to form particles that are readily engulfed by DPSC and can be controlled by magnetism. The carboxyl groups on the nGO surface provide binding sites for GFs, and BMP-2 was successfully integrated into the surface of the material, resulting in a significant increase in bone formation [101]. Similarly, Zhong et al. successfully integrated BMP-2 into nGO to treat osteoarthritic rats [102].

Metal-based nanoparticles have been applied in bone regeneration due to their outstanding antibacterial properties. Li et al. synthesized VEGF-loaded silver nanoparticles (Ag NPs) to improve aseptic necrosis after fracture surgery, and stimulated the proliferation of MSC to heal fracture wounds [103]. Similarly, a titanium dioxide nanoparticle was synthesized and bound to human morphogenetic protein to promote fracture healing as well as achieve antimicrobial function [104]. In another work, PDGF-BB was immobilized on the surface of titanium nanotubes (NTs) to utilize the synergistic effect of the surface morphology of NTs and PDGF-BB to promote BMSC proliferation [105].

Organic nanoparticles with high molecule-loading capacity have garnered significant attention from scientists, and liposome nanoparticles as well as polymeric nanoparticles are the two main categories [106]. Liposomes protect their core contents through a hydrated phospholipid bilayer and provide protection against both hydrophobic and hydrophilic drugs [107]. The use of liposome-loaded EGF has long been shown to promote bone regeneration in the rat alveolus [108], and there have also been many studies focusing on the delivery of BMP-2 in liposomes [109]. Li et al. synthesized a non-phospholipid liposome nanoparticle and loaded a smoothened agonist into the nanoparticle, which was then modified by coating the surface of the bone implant. RT-qPCR results showed a 19.8-fold upregulation of ALP expression in the cells after 7 days of co-culturing [110]. A liposome nanoparticle, modified with alendronate to target bone minerals and encapsulate the SDF-1 gene, promoted stem cell migration. In vivo experiments demonstrated that the systemically injected nanoparticles effectively targeted bone and reduced clearance by the kidney [111].

Natural polymers have been widely studied by scientists as they have a composition similar to the ECM in vivo and have excellent ability to mimic the physiological microenvironment of the skeleton [112]. For instance, an nHA-bound polylactic acid-polyethene glycol material has been shown to slow the release of BMP-2 for up to 21 days, leading to new bone fusion in a low dose (3 µg) of rat spinal defects [10]. Similarly, a poly (methyl methacrylate-co-methacrylic acid) nanoparticle with >80% loading efficiency was developed for BMP-2 delivery [113]. In addition, BMP-2 was encapsulated in poly (lactic-co-glycolic acid) (PLGA) nanoparticles and combined with alginate microcapsules loaded with VEGF to form a new delivery system. By slowly releasing GFs for over 1 month, this system achieved 82.3% new bone formation in rat cranial defects [114].

More recently, scientists have focused on combining liposomes with polymers. For example, a liposome with a sodium alginate and chitosan coating was used for EGF delivery, which slowed down the diffusion rate of EGF to achieve a slow release effect [115]. The soybean lecithin (SL)/BMP-2 complex was loaded into the water-filled nanopores of PLGA-based microspheres in the presence of SL and achieved an embedding rate close to 100%. Due to the high encapsulation rate, this material exhibited a slow release of GFs and promoted hBMSC differentiation [116].

Extracellular vesicles (EVs) are natural nanoscale materials secreted by cells that have emerged as promising alternatives to cellular therapy in bone repair [117]. These vesicles contain nucleic acids, proteins, lipids, and various signal molecules that are believed to have a positive effect on bone repair. Moreover, EVs are better able to avoid the immunogenic reactions associated with the direct use of cells [118]. Li et al. demonstrated the osteogenic potential of EVs secreted from adipose-, bone-marrow-, and synovial-derived MSCs in vitro. They loaded BMP-2 onto the EVs and subcutaneously injected them into nude mice. Adipose-derived EVs showed higher COL1 protein expression, indicating their excellent osteogenic capacity [119].

## 3. Endogenous Growth Factor Activation

The use of nanomaterials for bone repair has been a topic of research for many years, with an emphasis on using exogenous GFs to achieve synergistic bone regeneration; however, with advancements in medical technology, the focus has shifted to the activation of endogenous GFs, which can avoid the potential immunological side effects associated with exogenous bioactive molecules. Studies have explored using a patient’s own endogenous GF-rich fibrin, combined with silica nanofibers, to form an injectable hydrogel that demonstrated sustainable GF release and promoted osteoblast differentiation [120]. Platelet-rich plasma, due to its rich GF content, has also been used to modify nanofibers [121]; however, there are many different types of GFs in blood, and not all of them have a positive effect on bone repair in addition to their clinical manifestations not being clear [122]. Scientists have therefore focused on bone substitute materials that stimulate the release of endogenous GFs, making the use of nanomaterials a major direction of research. Nanomaterials can stimulate the production of endogenous GFs by releasing ions, activating signal pathways or the immune system, or regulating GF expression levels in the body through genetic engineering (Figure 1).

### 3.1. Signal Pathway Activation

Nanomaterials are increasingly being explored as a means by which to activate GFs in order to stimulate signal pathways and promote bone repair [42,123,124]. The release of ions or the loading of small-molecule drugs onto nanomaterials can activate GFs’ signal pathways, leading to enhanced bone regeneration. In recent years, studies have explored various types of nanomaterials and their impacts on endogenous GFs, demonstrating the potential for this approach to significantly improve bone repair outcomes. Overall, the use of nanomaterials to activate endogenous GFs and promote bone repair is an exciting area of research, with great potential to improve patient outcomes.

**Figure 4:**
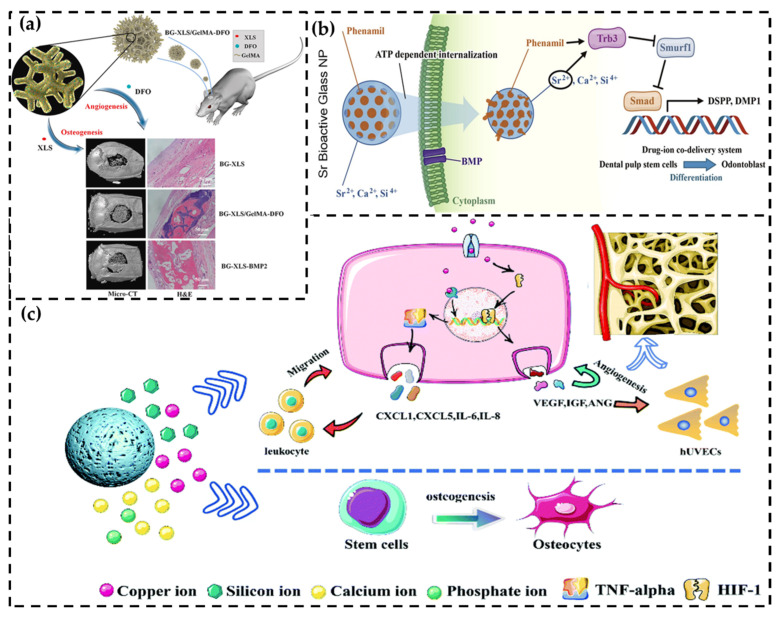
Illustration of the mechanism by which nanomaterials activate the signal pathway of GFs, leading to the stimulation of GF production. (**a**) GelMA-DFO attached to the BG-XLS stent allows for sustained release of DFO, which activates HIF-1α and thereby enhances osteogenesis [128]. (**b**) MBNs loaded with phenamil release both Sr ions and phenamil, stimulating the BMP-2 signal pathway [51]. (**c**) Cu-BG promoted bone regeneration by activating the HIF-1α as well as TNF-α pathways and releasing GFs [41].

The utilization of ions released from nanomaterials provides a unique and efficient way to stimulate signal pathways for GF production. This approach has demonstrated the potential to enhance cellular responses and promote bone regeneration. As shown in Figure 4c, the use of nano-bioactive glass (nBG) has been shown to effectively release copper ions and stimulate the HIF-1α as well as TNF-α pathways, leading to an increase in the release of endogenous VEGF, angiogenin, IGF-1, and TIMP. A significant increase in the expression of VEGF, as well as the excellent osteoinductivity and osteoconductivity, are demonstrated in a rat cranial defect model [41]. A separate study on nBG showed that the release of Ca^2+^ and SiO_4_^4–^ from nBG stimulates the release of VEGF and promotes angiogenesis through the activation of the PI3K/Akt/HIF-1α pathway [125]. In addition, MSNs modified with strontium ions were found to effectively stimulate the Wnt pathway, leading to an increase in VEGF production and promoting both osteogenesis as well as angiogenesis [126]. Nanomaterials have also been used as the surface coatings of bone substitutes to activate pathways. For example, nGO was coated onto the surface of titanium implants and found to activate the FAK/P38 signal pathway, resulting in the increased osteogenic differentiation of BMSCs [127]. Similarly, nHA was employed as a coating on biphasic CaP scaffolds, which enhanced the expression of the BMP-2 gene via the activation of the BMP/Smad signal pathway [13].

Nanomaterials have the potential to enhance the release of endogenous GFs through the delivery of small-molecule drugs that activate signal pathways. One such example is the use of desferrioxamine (DFO), an iron chelator that creates a hypoxic environment and activates the HIF-1α signal pathway. In a study, DFO was loaded into polylactic acid (PLA) nanospheres and transformed into nanofibrous membranes. The results showed that the use of DFO consistently elevated HIF-1α mRNA expression [43]. To further improve the longevity of small-molecule drugs, a novel nano-scaffold was introduced, as shown in Figure 4c. Specifically, a combination of DFO and GelMA was mixed and cross-linked on a bioglass scaffold functionalized with nanoclay (BG-XLS) to achieve the sustained release of DFO for up to 21 days. ELISA results revealed the high expression of two GFs, HIF-1α and VEGF, and endogenous bone regeneration was also observed in a rat cranial defect model [128]. Furthermore, nanomaterials have the potential to stimulate multiple signal pathways simultaneously through ion stimulation and loading with small-molecule drugs. As depicted in Figure 4b, a mesoporous bioglass nanoparticle (MBN) modified by Sr ions was loaded with phenamil, an activator of the BMP signal pathway. The combined effect of the Sr ions and phenamil accelerated the degradation of Smurf, a BMP pathway antagonist, leading to the upregulation of SMAD1/5/8, the stimulation of the BMP pathway, and an increase in the production of endogenous GFs [51]. Additionally, EVs secreted by genetically modified MSCs that consistently express the BMP-2 protein were used for bone repair. These EVs were shown to enhance the BMP-2 signal pathway of hMSCs, leading to the stimulation of the secretion of endogenous growth factors and resulting in bone regeneration in vivo in a rat cranial defect model [129].

### 3.2. Immune System Stimulation

In the complex process of bone repair, a variety of cell types are involved, including the body’s immune cells, which play a crucial role in the initial inflammatory response that initiates the healing of the bone defect. Immune cells are capable of regulating bone homeostasis by producing various endogenous GFs, such as TGF-β and IL-4 [29], to stimulate bone repair. Hence, immune cells have become a target for researchers seeking to stimulate the production of endogenous GFs. It is worth noting that, upon entering the body, nanomaterials are often initially absorbed by the phagocytes of the immune system [130], providing a basis for their ability to stimulate the immune system. A variety of immune cells (e.g., macrophage, monocytes, and T cells) have been shown to activate and release endogenous GFs through ions carried by nanomaterials, small-molecule drugs, or the surface topology of materials.

#### 3.2.1. Macrophage

Macrophages play a vital role in bone regeneration by removing dead cells and releasing GFs during pre-osteogenesis to control inflammation and promote osteogenesis. They are divided into two phenotypes, M1 and M2 types, both of which are essential in bone repair. During the early osteogenesis, M1 macrophages secrete cytokines, such as IL-6, IL-1, and TNF, to recruit stem cells and promote bone repair [28], while M2 macrophages produce GFs such as VEGF and PDGF to stimulate blood vessel formation as well as bone regeneration in late osteogenesis [46]. The transition from one phenotype to another is called macrophage polarization. As macrophage polarization and GF secretion play significant roles in osteogenesis, researchers are interested in inducing macrophage polarization and maximizing GF secretion for improved bone repair. By inducing macrophage polarization and boosting the production of GFs, nanomaterials have shown great potential in enhancing bone repair.

**Figure 5:**
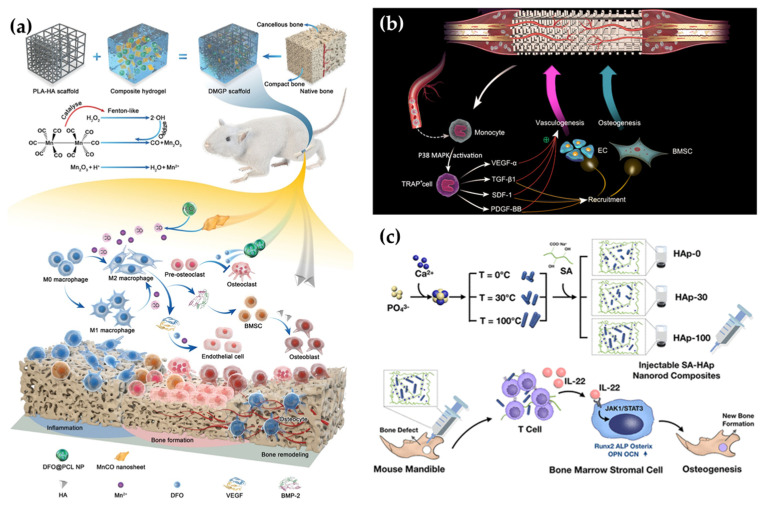
Activation of the immune system by nanomaterials to release GFs. (**a**) A composite hydrogel containing MnCO nanosheets that release CO and stimulate macrophage polarization, resulting in the release of GFs [52]. (**b**) Scaffolds loaded with zinc silicate nanocrystals differentiate monocytes into TRAP^+^ cells, which express higher levels of GFs [138]. (**c**) Hydroxyapatite nanorods induce T cells to produce IL-22 [14].

Several studies have shown that nHA can introduce the polarization of human macrophages to the M2 phenotype and promote the osteogenic differentiation of BMSC cells. Mahon et al. found that nHA treatment increased M2 macrophage markers, and the nanomaterial-treated macrophage medium was able to promote the osteogenic differentiation of BMSC cells. An elevated level of IL-10 was observed in rats implanted with the material [131]. In another study, an nHA-mineralized collagen scaffold (HIMC) that mimics the natural bone structure was found to introduce the polarization of macrophages to the M2 phenotype, resulting in the release of IL-10 [132]. Additionally, a chitosan/agarose/nHA scaffold was shown to stimulate M2 macrophages’ polarization and release of GFs, which promoted the osteogenic differentiation of BMSC cells [133]. In addition to nHA, gold-based nanoparticles have also been reported to modulate the immune system. Liang et al. utilized the porous structure of MSNs to transport gold nanoparticles, which were found to have low cytotoxicity. The material effectively down-regulated the number of M1-type macrophages and up-regulated the BMP-2, TGF-β1, and VEGF genes [134]. A liposomal nanoparticle wrapped around a titanium matrix has also been shown to promote M2 polarization in macrophages, thereby promoting bone repair [135]. Other studies have also found that EVs secreted by intravenous MSCs can stimulate macrophage polarization to the M2 phenotype and enhance the secretion of endogenous TGF-β [136]. Apart from the direct stimulating effect of material on macrophages, recent research has revealed that nanomaterials can also interact with endogenous substances to induce macrophage polarization. A recent study utilized a hydrogel scaffold that incorporated a MnCO nanosheet loaded with poly(ε-caprolactone) nanoparticles containing DFO (Figure 5a). MnCO reacted with endogenous hydrogen peroxide at the bone defect site, resulting in the generation of CO and Mn^2+^. This upregulated the expression of M2 macrophages and promoted endogenous VEGF and BMP-2 release, while DFO was able to inhibit osteoclastogenesis [52]. In addition, the polarization of M1 macrophages is also being investigated. A copper-coated titanium substrate (Cu-Hier-Ti surface) with micro–nano features was found to promote M1 polarization by releasing copper ions and demonstrate osteogenic capacity [137]. The titanium coating also has strong antibacterial properties, making the material well suited for implantation to reduce bacterial infection at the site of injury.

Nanomaterials can also be used as carriers for biomolecules that act as polarizers. For instance, resolvin D1 (RvD1) was loaded as an M2 activator in a gold nanocage (AuNC), and its release was controlled by near-infrared light, leading to the stimulation of M2 macrophages’ polarization and the promotion of bone repair [139]. A TiO_2_ nanotube, coated with dopamine and functionalized with IL-4 and RGD peptide, was used to stimulate the switch of macrophages to the M2 phenotype and enhance osteogenesis by mimicking the extracellular matrix [140].

In summary, recent studies have focused on either M1 or M2 macrophages, but both are important in osteogenesis. It is crucial to polarize macrophages to a specific phenotype at a specific time for effective endogenous bone repair, and this is an area for future research.

#### 3.2.2. Monocytes and T Cells

Scientists have discovered that activated monocytes and T cells can also release endogenous GFs, in addition to macrophages. Monocytes are a type of white blood cell that directly regulate the immune response and can produce various GFs to stimulate osteogenic gene expression when activated. A scaffold of nHA, zinc silicate, and collagen stimulated monocytes’ differentiation into tartrate-resistant acid phosphatase-positive (TRAP+) cells, which are pro-osteoclasts that release GFs to induce bone regeneration. This study found that SDF-1, TGF-β1, VEGF-α, and PDGF-BB expression was elevated, leading to the significant promotion of angiogenesis, stem cell homing, and osteogenic differentiation [13]. In addition, nGO has also been shown to induce monocyte activation. Bordoni et al. used GO in a complex with CaP to activate monocytes, resulting in the production of oncostatin M, an osteoinductive factor that stimulates bone formation in mice tibia [141].

T cells are a vital component of the immune system and can secrete endogenous GFs upon activation. As shown in Figure 5c, biomimetic HA nanorods with different aspect ratios were synthesized to stimulate T cells for IL-22 release. The results showed that nanorods with a high aspect ratio had a greater osteogenic capacity and lead to a significant increase in T cells [14]. Moreover, a titanium dioxide nanotube with a defined diameter (105 nm) was shown to stimulate T cells and release FGF-2, which promotes the proliferation of BMSCs [142].

Taken together, the shape of a nanomaterial and its surface morphology play a crucial role in activating T cells to secrete GFs. While most studies on stimulating the bone immune system have focused on macrophage polarization in innate immunity, there is a shortage of research on adaptive immunity, emphasizing the need for investigations into monocytes and T-cell-based immune responses.

### 3.3. Gene Therapy

Gene therapy is an emerging therapeutic approach in molecular biology, aimed at treating or preventing genetic diseases by introducing or modifying genes in a patient’s cells. Gene therapy can be used to regulate gene expression in the body and treat a range of diseases. Gene therapy can also provide a long-term effect with a single administration, and has the potential to reduce or eliminate the need for repeated drug administration [143]. In the field of bone repair, gene therapies using synthetic RNAs and plasmids have been used to enhance the expression of osteogenic GFs in vivo; however, due to the degradation of these gene drugs by enzymes in the body and their inability to be effectively internalized by target cells [144], their use has been inefficient. To address this challenge, scientists are exploring the use of nanomaterials as carriers for gene drugs to protect them for safe and efficient transport into cells.

#### 3.3.1. RNA

RNA therapy is an advanced field in contemporary gene therapy; in this section we will discuss the regulation of bone repair by three different types of RNAs (microRNA, small interfering RNA, and messenger RNA). MicroRNA, also known as miRNA, is a small single-stranded RNA that binds to target mRNAs and achieves regulatory effects by repressing post-transcriptional gene expression. It has been shown to regulate the endogenous expression of a variety of GFs [145]. miRNAs are capable of being loaded onto many different forms of nanomaterials, such as nanocapsules, hygrogels, and scaffolds. miR-21 nanocapsules were prepared using in situ polymerization, bound to CMCS into a hydrogel, and used as a nano-coating. miR-21 was able to promote bone repair via the PI3K/β-catenin pathway, and the coating was shown to promote CD31 expression as well as enhance angiogenesis [146]. Tetrahedral DNA nanostructures (TDNs) were incorporated into lithium heparin hydrogels to deliver miR335-5p, targeting the osteonecrosis-inducing signal Dickkopf-1 (DKK1) and upregulating the Wnt pathway to stimulate endogenous VEGF. Western blot and immunofluorescence results showed that VEGF secretion significantly elevated while decreasing DKK1 expression, and a high level of new bone and blood vessel production was also observed in rabbits [147]. A scaffold containing chitosan (CS), nano-hydroxyapatite (nHAp), and nano-zirconium dioxide (nZrO2) was prepared for the delivery of miR-590-5p. miR-590-5p could target Smad7 and enhance the expression of Runx2, while Zr ions stimulated the BMP pathway. In vitro results showed that the scaffold could enhance the secretion of Runx2, ALP, and the gene expression of Col1 [11]. EVs, as natural miRNA-rich nanomaterials, have become one of the research directions through which scientists can explore the miRNAs that can promote the production of endogenous GFs [148]. Guo et al. identified miR-206-3p from EVs secreted by orofacial mesenchymal stem cells (OMSCs) and demonstrated that it significantly increased BMP-3 expression in addition to enhancing osteogenic activity [149]. EVs secreted by cells under different environmental stimulations have also been shown to exert different effects. EVs secreted by BMP-2-stimulated macrophages were shown to significantly increase the secretion of GFs such as FGF when integrated into the surface of titanium nanotubes, but the mechanism is unclear; the authors attribute this to the miRNA component of the EVs [150]. Zhuang et al. found that EVs secreted by hypoxia-treated MSCs contained miR-210-3p, which promoted endogenous HIF-1α secretion, and observed vascularized bone regeneration in rat cranial defects [151]. In addition, artificially engineered EVs were also available. EVs secreted by the lentiviral transfection of hASCs overexpressing miR-375 were used for miRNA regulation. the introduction of EVs resulted in a significant enhancement of the expression of the positive regulator of osteogenesis, miR-375, in cells, and significantly enhanced the expression of the osteogenesis-related genes RUNX2, ALP, COL1A1, and OCN in in vitro experiments [152].

In addition to loading miRNAs, nanomaterials can also modulate the expression of endogenous GFs by loading miRNA inhibitors. Building on the ability of miR-214 to target ATF-4 and thereby affect bone regeneration [153], Ou et al. loaded a PEI-modified GO with a miR-214 inhibitor to target and inhibit miR-241, activate ATF-4, and enhance endogenous GF production. After treatment with the material, miR-241 levels were significantly downregulated, and ATF-4 as well as OCN protein levels were significantly increased. The material was implanted into rat cranial defects after incorporation into the scaffold, and the defects were barely observed in microCT after 16 weeks [154]. Other studies have shown that miRNA stimulated the secretion of endogenous GFs through alternative pathways. For instance, the co-delivery of miRNAs and GFs can induce the differentiation of T cells into Treg cells. In a nanofiber sponge microsphere (NF-SMS) containing PLGA loaded with miR-10a and MSNs containing IL-2/ TGF-β, all three biologics were able to achieve a slow release for more than twenty days, and the activation of functionalized Treg cells was observed by flow cytometry. In vitro and in vivo results also showed the downregulation of effector-T-cell-associated cytokines, the enhancement of osteoblast activity, and the inhibition of osteoclast activity through Treg cell activation [155].

**Figure 6:**
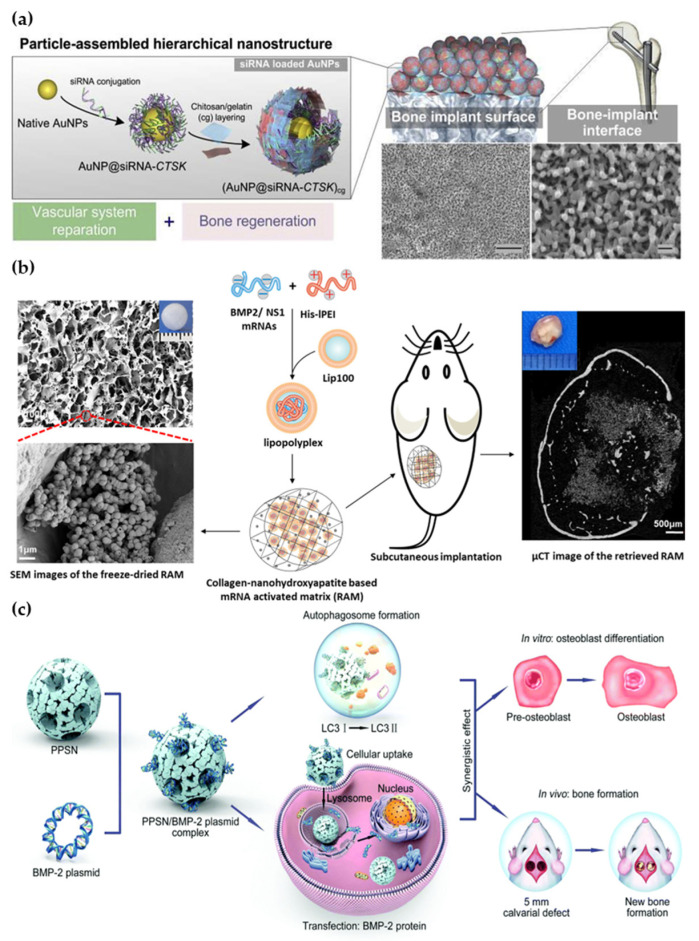
Nanomaterial-assisted genetic technology for endogenous GF modulation. (**a**) siRNA-decorated AuNPs facilitated the release of GFs by regulating transcription [156]. (**b**) A dual system using BMP2/NS1 mRNAs loaded nano-lipopolyplexes for the prolonged expression of GFs and inducing new osteogenesis [53]. (**c**) Encapsulating the BMP2 plasmid by PPSNs increased the expression of GFs and promoted osteogenic differentiation [50].

Although small interfering RNA (siRNA) and miRNA are both short RNAs, siRNA has a double-stranded structure and is able to cleave the target gene mRNA to achieve gene silencing specifically. As shown in Figure 6a, siRNA was modified on gold nanoparticles (AuNPs), which were attached to the bone implant as a coating. The gold nanoparticles were internalized by macrophages and provided up to eight days of slow release to protect and deliver siRNA, which effectively inhibited cathepsin K (CTSK), highly expressed in osteoblasts, and genetically enhanced endogenous PDGF-BB and VEGF release [156]. Messenger RNA (mRNA) is a single-stranded RNA that carries genetic information that is transcribed from DNA. Through translation, mRNA is able to form proteins with corresponding functions. Thus, by introducing mRNA the body can produce GFs on its own, thereby promoting bone repair; however, exogenous mRNA can cause immune rejection in the body, so Wang et al. used an NS1 mRNA, which could inhibit the human RNA sensor, in combination with BMP-2 mRNA, delivered via a nanomaterial carrier. As shown in Figure 6b, the NS1/BMP-2 mRNA complex was loaded into a liposome (LPR) and used to significantly enhanced BMP-2 production in hMSCs. The LPR was then loaded onto a collagen-hydroxyapatite scaffold for in vivo implantation. The results showed that mice implanted with the LRP scaffold had 2.1-fold more new bone production than mice implanted with a blank scaffold eight weeks after implantation [53].

#### 3.3.2. Plasmid

A plasmid is a type of DNA that is artificially encoded to confer the ability to express a specific protein. Scientists have been able to increase the levels of GFs secreted by cells by translocating plasmids of GFs into cells; however, plasmids are often degraded in lysosomes, which reduces their efficiency in entering the nucleus. To address this challenge, nanomaterials are often used as delivery vehicles for plasmids due to their high biocompatibility and protective properties. Several studies have reported the use of nanomaterials in delivering the BMP-2 plasmid (pBMP-2), which is a key GF in the bone repair process. As shown in Figure 6c, the pBMP-2 was loaded into polyethyleneimine-modified porous silica nanoparticles (PPSNs) and significantly promoted cellular BMP-2 secretion [50]. Similarly, the nanocomplex formed by binding pBMP-2 and chitosan was immobilized on a scaffold for bone repair [157]. In another study, pBMP-2 was bound to CaP to prolong the release of BMP-2 [158].

To enhance the transfection efficiency of the plasmid, scientists used different modifications of the nanomaterials. Jalal and Dixon used glycosaminoglycan (GAG)-binding enhanced transduction (GET) peptides as the surface moiety of pBMP-2-encapsulated PLGA nanoparticles to enhance their cell penetration and achieve higher transfection efficiency by five orders of magnitude [159]. In addition to pBMP-2, the plasmid encoding VEGF, the most common GF that promotes angiogenesis, has been shown to promote bone repair. To further explore the potential of this growth factor, a nano-complex of PEI with pVEGF was developed and bound to a PDA-modified PLA scaffold. This scaffold achieved an encapsulation efficiency of 60% and a sustained release of 144 h [160]. This innovative approach presents promising opportunities for promoting bone repair through the sustained delivery of VEGF. The incorporation of exosomes derived from a chondrocyte line, ATDC5, carrying a VEGF plasmid, was achieved through 3D printing. They were successfully transfected into cells after 48 h, and subsequent analyses using PCR and ELISA results indicated a significant increase in intracellular VEGF content [161]. In addition to BMP-2 and VEGF, TGF-β1 is a crucial growth factor involved in the osteogenesis process. CuS nanoparticles loaded with pTGF-β1 exhibited highly efficient transfection compared to commercial transfectants. Furthermore, these nanoparticles were found to promote stem cell migration for chondrogenesis and were able to secrete TGF-β1, making them promising candidates for in vivo osteoarthritis treatment [162]. In addition, EVs have also been used to encapsulate plasmids. In the work conducted by Chen et al., EVs secreted by ATDC5 were used to encapsulate pVEGF. The combination of EVs and a 3D-printed scaffold resulted in a significant increase in angiogenesis and new bone production in vivo [152].

As research progressed, scientists have found that the delivery of individual plasmids alone may have limitations in promoting bone repair [163]. As a result, the combination of multiple plasmids is slowly being put into practice. Currently, the most commonly used combination for bone repair is pBMP-2 and pVEGF. An organic nanomaterial, star-shaped poly(L-lysine) polypeptides (star-PLLs), has been utilized to load BMP-2 and VEGF plasmids in order to form nanodrugs. The resulting nanodrugs are capable of promoting GF production and have been shown to support rapid bone repair in a rat cranial defect model within four weeks [164]. In order to improve the efficiency of plasmid entry into the nucleus, a cell-penetrating peptide, KALA, was used to modify PLGA/PEI nanoparticles for the dual delivery of pBMP-2 and pVEGF. To enable sustained release for more than 21 days, the nanoparticle was encapsulated in a fibrous hydrogel [165]. The combination of BMP-2 and PDGF-B for bone repair has also been reported. Moncal et al. developed a 3D-printed scaffold incorporating pPDGF-B and chitosan-nanoparticle-encapsulated pBMP-2 to slow the release of plasmids for forty-two days. The nanomaterials provided protection for the plasmids, and the expression level of rBMSC-associated GFs was found to have significantly increased within two weeks [166].

## 4. Conclusions and Future Perspectives

In conclusion, the use of nanomaterials for the delivery and activation of GFs has shown great potential in promoting bone repair. By using various types of nanomaterials, including scaffolds, hydrogels, nanofibers, and other forms of nanomaterial implants, the controlled delivery of exogenous GFs has been achieved, allowing for the sustained expression of these factors over an extended period of time. Moreover, by leveraging the unique properties of nanomaterials, such as high surface areas and tunable physicochemical properties, researchers have been able to improve the bioavailability as well as bioactivity of GFs for more efficient bone regeneration. In addition, scientists are exploring the possibility of adding more biological properties to nanomaterials to facilitate the bone repair process, such as antibacterial, anti-inflammatory, and mechanical properties. Besides delivering exogenous GFs, nanomaterials have also been successfully used to modulate endogenous GFs by activating signal pathways, stimulating the immune system, and enabling gene therapy. This approach avoids the potential immunological side effects associated with exogenous bioactive molecules while harnessing a patient’s own regenerative ability, offering a promising future for personalized medicine.

Looking to the future, further research is needed to optimize the interaction between nanomaterials and GFs for bone tissue engineering. Specifically, efforts should be made to improve the efficiency of transfection and reduce the potential toxicity of nanomaterials. Moreover, there is a need for more long-term studies that investigate the safety as well as efficacy of these systems in clinical settings. Finally, the development of personalized medicine approaches that tailor the choice of growth factors and delivery systems to individual patients holds great potential for improving the outcomes of bone repair therapies.

## Data Availability

Data sharing not applicable.
